# Development of an Optimized Process for Functional Recombinant SARS-CoV-2 Spike S1 Receptor-Binding Domain Protein Produced in the Baculovirus Expression Vector System

**DOI:** 10.3390/tropicalmed8110501

**Published:** 2023-11-16

**Authors:** Mohamed Boumaiza, Ameni Chaabene, Ines Akrouti, Meriem Ben Zakour, Hana Askri, Said Salhi, Wafa Ben Hamouda, Soumaya Marzouki, Chaouki Benabdessalem, Melika Ben Ahmed, Khaled Trabelsi, Samia Rourou

**Affiliations:** 1 Laboratory of Molecular Microbiology, Vaccinology and Biotechnology Development, Group of Biotechnology Development, Institut Pasteur de Tunis, Université Tunis El Manar, Tunis 1002, Tunisia; 2 Laboratory of Transmission, Control and Immunobiology of Infections (LTCII), LR11IPT-02, Institut Pasteur de Tunis, Université Tunis El Manar, 13, Place Pasteur. BP. 74, Tunis 1002, Tunisia

**Keywords:** multiplicity of infection, cell density at infection, SARS-CoV-2, spike S1 glycoprotein, receptor-binding domain, Sf9 cells

## Abstract

To map the spread of Severe Acute Respiratory Syndrome Coronavirus 2 (SARS-CoV-2) and evaluate immune response variations against this virus, it is essential to set up efficient serological tests locally. The SARS-CoV-2 immunogenic proteins were very expensive and not affordable for lower- middle-income countries (LMICs). For this purpose, the commonly used antigen, receptor-binding domain (RBD) of spike S1 protein (S1RBD), was produced using the baculovirus expression vector system (BEVS). In the current study, the expression of S1RBD was monitored using Western blot under different culture conditions. Different parameters were studied: the multiplicity of infection (MOI), cell density at infection, and harvest time. Hence, optimal conditions for efficient S1RBD production were identified: MOI 3; cell density at infection 2–3 × 10^6^ cells/mL; and time post-infection (tPI or harvest time) of 72 h and 72–96 h, successively, for expression in shake flasks and a 7L bioreactor. A high production yield of S1RBD varying between 4 mg and 70 mg per liter of crude cell culture supernatant was achieved, respectively, in the shake flasks and 7L bioreactor. Moreover, the produced S1RBD showed an excellent antigenicity potential against COVID-19 (Wuhan strain) patient sera evaluated by Western blot. Thus, additional serological assays, such as in-house ELISA and seroprevalence studies based on the purified S1RDB, were developed.

## 1. Introduction

SARS-CoV-2, responsible for coronavirus disease 2019 (COVID-19), is an enveloped virus belonging to the betacoronavirus group of the Coronaviridae family. Its large single positively stranded RNA of 29,891 nucleotides and 9860 amino acids encodes four major structural proteins: the spike (S), envelope (E), membrane (M), and nucleocapsid (N) proteins [1]. To date, there is only one specific oral treatment for COVID-19, Paxlovid, a ritonavir-boosted nirmatrelvir that was approved by the Food and Drug Administration (FDA) on 25 May 2023 [2]. Despite the newly commercialized vaccines being relatively quickly developed, due to the emergency that the situation imposes, the virus continues its propagation globally. The strategies used for all commercial vaccines have been based on the immunogenic potential of the trimeric spike glycoprotein (S), composed of two subunits, S1 and S2 [3]. The S1 subunit contains the receptor-binding domain (RBD), essential for binding to the peptidase domain of angiotensin-converting enzyme 2 (ACE2). The latter is widely expressed in various human organs including the lungs, heart, brain, and kidneys [4]. Thereby, it plays an essential role in virus attachment, fusion, and entry into the host cell [5]. On the other hand, the S2 subunit is involved in the fusion of the virus and the host cell membranes [6,7,8,9]. Thanks to genome sequencing, scientists discovered the emergence of new variants due to different mutations at the spike protein level [10,11]. Indeed, the RBD region, within the spike S1 subunit, is the cornerstone of the virus for its spread through infected cells containing the ACE2 receptor [12]. Therefore, RBD-based vaccines and diagnostic approaches have been extensively developed [5,13,14]. For example, four versions of the spike (S1) protein of SARS-CoV-2 were expressed in Sf9 insect cells, including the RBD. They showed excellent antigenicity against convalescent COVID-19 patient sera through ELISA and elicited a high neutralization titer in mice [14]. This prompted us to further study the BEVS process parameters for optimized RBD production. Some influent parameters such as the time of post-infection (tPI), cell density at infection, and multiplicity of infection (MOI) were concerned. Sf9 insect cells grown on a commercial animal-component-free medium (SF900II) were used. In fact, BEVS, first described in the early 1980s, has been successfully used for the production of various recombinant proteins [15]. During this study, we investigated the expression of the RBD (His-tagged) of the SARS-CoV-2 S1 subunit (S1RBD, Wuhan strain) under different culture conditions. The upstream and downstream process parameters were optimized. This may be of utmost interest for the production of several recombinant proteins with therapeutic and diagnostic potential.

## 2. Materials and Methods

### 2.1. Upstream Processing

#### 2.1.1. Construction of the Recombinant pFastBac-S1RBD and Bacmid Generation

The RBD cDNA sequence (residues 319–541) of the SARS-CoV-2 spike S1 subunit, Wuhan strain (GenBank accession number: QHD43416.1), was cloned into the baculovirus shuttle, pFastBac/CT-TOPO vector (Invitrogen, Waltham, MA, USA), downstream to the gp67 signal sequence and a His-tag, in the C-terminal extremity. In fact, the RBD constructs were fused with an N-terminal gp67 signal peptide and a C-terminal His6 tag to facilitate extracellular secretion and permit affinity purification, respectively (Figure S1). The recombinant pFASTBac-S1RBD vector was kindly provided by Chris KP Mok from the HKU-Pasteur Research Pole, Li Ka Shing Faculty of Medicine, University of Hong Kong, China [16]. The generation of recombinant bacmid DNA, containing the RBD sequence, was performed using the Bac-to-Bac C-His TOPO expression system kit following the manual user guidelines (Invitrogen). Briefly, DH10Bac Competent Cells (Invitrogen) were transformed with the recombinant pFastBac-RBD plasmid to generate the recombinant bacmid DNA by homologous recombination. Then, twelve white colonies (on LB agar plates containing: 50 µg/mL of Kanamycin, 7 µg/mL of gentamicin, 10 µg/mL of tetracycline, 100 µg/mL of X-Gal, and 40 µg/mL of IPTG) were screened by PCR. pUC/M13 Forward: 5′-CCCAGTCACGACGTTGTAAAACG-3′ and pUC/M13 Reverse: 5′-AGCGGATAACAATTTCACACAGG-3′ primers were used. The PCR product was visualized on a 1.5% agarose gel stained with ethidium bromide. Finally, the recombinant bacmid was isolated using the PureLink HiPure Plasmid DNA Miniprep Kit (Invitrogen).

#### 2.1.2. Insect Cell Culture

The Sf9 insect cell line, a cell line derived from the pupal ovary tissue of the Fall Armyworm (*Spodoptera frugiperda*) was used in this study. The Sf9 cells were grown in suspension on SF900II serum-free medium (Gibco, Billings, MT, USA, catalog number 11496-015) without antibiotics addition in polycarbonate Erlenmeyer flasks (Corning) at 27 °C and 115–120 rpm. The seeding cell density was 0.5 × 10^6^ cells/mL, and the sub-cultivation frequency was twice a week.

#### 2.1.3. Transfection

The Sf9 cells were maintained in the exponential growth phase, at a cell density of 1.5–2.5 × 10^6^ cells/mL and viability higher than 95%, in 25 cm^2^ culture flasks containing a total of 2 × 10^6^ cells at a final volume of 5 mL. Twenty µL of ExpiFectamine Sf transfection reagent was diluted in 500 µL of the medium in a sterile Eppendorf tube. Then, 2 µg of the recombinant bacmid DNA was added. The mixture was inverted 10 times and incubated for 5 min at room temperature to form a DNA–lipid complex. Transfected cells were incubated at 27 °C for 4–5 days to generate the recombinant baculoviruses (Figure S2). Untransfected cells cultivated under the same conditions served as a control.

#### 2.1.4. Amplification of Baculovirus Stocks

When the viability fell to 70–80%, the transfected cells were harvested by centrifugation at 3500× *g* for 10 min, and the supernatant containing the recombinant baculoviruses (P0 stock) was stored at 4 °C (stable for 6 months) or at −80 °C (long-term storage). The recombinant P0 baculovirus stock was amplified to obtain a high titer of recombinant virus by adding 0.5 mL of P0 to 20 mL of Sf9 cells (2 × 10^6^ cells/mL) and incubating for 2–4 days until reaching viability between 70 and 80%. The cells were harvested and the culture supernatant, containing the recombinant baculoviruses (P1 stock), was stored at 4 °C for further baculovirus amplification as described in Figure S3.

#### 2.1.5. Baculovirus Titration Using a Cell Viability Assay (TCID50)

The tissue culture infective dose (TCID50) method was improved by Mena et al. [17]. In this method, viral titration was performed by measuring cell viability based on the cleavage of the tetrazolium ring of 3-(4, 5-dimethylthiazol-2-yl)-2, 5-diphenyl tetrazolium bromide (MTT) by mitochondrial dehydrogenase in viable cells [18]. A magenta coloration results from that reaction and allows the quantification of viable cells via spectrophotometry. Sf9 cells were grown in the Sf900 II medium to reach a cell density of 3 × 105 cells/mL. Gentamicin was added to the cell culture at a concentration of 0.05% (*v*/*v*) to avoid any contamination. Then, 50µL per well of cell culture was added. The virus was diluted from 10-1 to 10-10 in another 96-well plate containing 90µL of medium per well. In fact, serial dilutions (10 µL virus in 90µL medium) were performed from lines 2 to 11. The plate was incubated at 27 °C for 6 days. After that, 10µL of MTT solution (5 g/L) was added to each well, and the plate was incubated at 27 °C for 2 h under gentle shaking. Afterward, the plates were centrifuged at 2000× *g* for 10 min, and the supernatant was discarded with a multi-channel pipet. After solubilization of the magenta salt crystals with Dimethyl-sulfoxyde (DMSO), the absorbance was measured at 570 nm with a microplate reader. To calculate the titer (plaque-forming units: pfu/mL), the data were analyzed using SigmaPlot 12.0 software.

#### 2.1.6. Production of S1RBD in Shake Flasks

Recombinant baculovirus, P1 stock, was used to infect the Sf9 cells, and the protein expression was monitored by Western blot analysis at different time points post-infection (0 h, 24, 72, 96, and 120 h) using two different multiplicities of infection (MOI 1 and 5). Furthermore, protein expression was analyzed at additional MOI values (0.01, 0.5, 1, 3, 5, 7, and 10) in the Sf9 cells using a cell density at infection of 2 × 10^6^ cells/mL and viability higher than 95% at 27 °C for 72 h post-infection with shaking at 110 rpm. S1RBD protein expression was also analyzed using different cell densities at infection (1 × 10^6^, 2 × 10^6^, 3 × 10^6^, 4 × 10^6^, 5 × 10^6^, and 7 × 10^6^ cells/mL) at an MOI of three.

#### 2.1.7. Production of S1RBD in 7L Controlled Bioreactors

Large-scale production of S1RBD was carried out in a 7 L bioreactor (BioBraun, Melsungen, Germany) as described by Rourou et al. [19]. It was equipped with a marine impeller and a spin filter (22 µm) fixed to the axis. The following conditions were maintained during both cell proliferation and infection phases: no pH regulation by CO_2_ sparging or addition of NaHCO_3_ at 88 g/L, and the dissolved oxygen was adjusted to 50% air saturation by continuous surface aeration. The temperature was maintained at 27 °C and the agitation rate at 125 rpm. The batch culture mode was started with a seeding cell density of 5 × 10^5^ cells/mL. When the cell density reached 4 × 10^6^ cells/mL, the cells were infected with recombinant baculovirus (titer 1.085 × 10^9^ pfu/mL) at an MOI of three. After the cell infection, samples were taken daily to determine cell density, cell viability, osmolarity, and S1RBD expression level.

### 2.2. Downstream Processing

#### 2.2.1. Tangential Flow Filtration (TFF)

The infected Sf9 culture supernatants (from the Erlenmeyer flasks or the bioreactor) were harvested by centrifugation at 4500 rpm for 20 min at 4 °C. The culture supernatants were then pre-treated by filtration at a flow rate of 20 mL/min through an MF-Millipore membrane filter, 8µm pore size (Millipore, Burlington, MA, USA, catalog number SCWP14250). Afterward, the clarified supernatants were concentrated and buffer-exchanged by TFF using a Cogent Microscale System (Merck Millipore, Burlington, MA, USA) as follows: A Master-Flex peristaltic pump fed the supernatant to a 10 KDa molecular weight cutoff cassette. Two references were tested, respectively, for the small-scale (maximum volume 500 mL) and medium-scale (volumes between 500 mL and 5 L) experiments; Pellicon XL 50 cm^2^ (Biomax Media; catalog number PXP010A50; Millipore) and Pellicon 3/0.11 2 (Ultracel membrane; catalog number P3C010C01; Millipore). The pre-treated supernatant was first concentrated to 50% of the initial volume and then buffer-exchanged with 4 to 5 volumes of 20 mM of PBS, 0.5 M of NaCl, and 20 mM of imidazole, pH 7.4. Subsequently, the concentration to the desired level was performed. Finally, the TFF cassette was rinsed, regenerated, and stored according to manufacturer instructions.

#### 2.2.2. Protein Purification

Recombinant His-tagged RBD spike S1 (S1RBD) protein was purified from the TFF retentate based on immobilized metal ion affinity chromatography using a HisTrap FF 5 mL column (catalog number 17524801; GE Healthcare, Chicago, IL, USA), following the manufacturer’s procedure, by the AKTÄ purifier system (GE Healthcare Life Sciences, Uppsala, Sweden). Prior to loading, the samples should be filtrated through 0.22 µm low-protein binding filters. We used Sarto Scale 25 (Sartorius, Göttingen, Germany, catalog number 5235307HV-LX-C) and Sartopore Platinium Capsules (single use, Sartorius, catalog number 5491307H4-SO-B), respectively, for the small-scale (maximum 200 mL) and medium-scale (200 mL to 1 L) assays. The ultra-low binding filters were kindly provided by Sartorius, Stedim Biotech, GmbH. The column was first equilibrated with at least 5 column volumes of the binding buffer (20 mM of PBS, 0.5 M of NaCl, and 20 mM of imidazole, pH 7.4). Then, the TFF-treated culture supernatant was applied to the column at a flow rate of 1 mL/min. The flow rate used at all the other purification steps was 5 mL/min. Afterward, the column was washed with a minimum of 10 CV binding buffer. The elution of the S1RBD was carried out using the elution buffer (20 mM of PBS, 0.5 M of NaCl, and 500 mM of imidazole, pH 7.4). Elution fractions containing the purified S1RBD were pooled, concentrated, and buffer-exchanged with a Vivaspin device 10 KDa (Sartorius). PD10 desalting columns (GE Healthcare) were also tested for buffer exchange (imidazole elimination). A second purification step, using size exclusion chromatography, was performed on a Superdex 200 Increase 10/300 GL column (GE Healthcare, catalog number 28990944) according to the manufacturer’s instructions. The equilibration and elution buffer was 20 mM of PBS containing 0.5 M of NaCl, pH 7.4. A 0.5 mL protein sample was injected through a 2 mL sample loop. Separation was performed at a flow rate of 0.75 mL/min. The eluate was collected into 1 mL fractions using a fraction collector. Protein concentrations of the purified and non-purified samples were determined according to the Bradford micro-assay protocol [20] with bovine serum albumin (BSA) as standard, using the Quick Start Bradford reagent (Bio-Rad, Hercules, CA, USA).

#### 2.2.3. SDS-PAGE

The protein fractions were analyzed by SDS-PAGE 4–12%, Bis-Tris, 0.75 mm, 12-well mini protein gel (Biorad, Hercules, CA, USA) under reduced (+5% β-mercaptoethanol) and unreduced (without β-mercaptoethanol) conditions to monitor protein size, purity, and dimerization. The gels were visualized by the silver-staining method using a ProteoSilver Stain Kit (Sigma-Aldrich, St. Louis, MO, USA) according to the manufacturer’s instructions. Visualization using Coomassie Brilliant Blue G-250 (BioRad) was also performed.

#### 2.2.4. Western Blot Analysis

Samples of 10 µg of purified S1RBD were analyzed on a 12% SDS polyacrylamide gel using the Bio-Rad Mini Trans-Blot cell system. The proteins were transferred to a nitrocellulose membrane (GE Healthcare). The membrane was first saturated using a blocking buffer (Phosphate-Buffered Saline: 137 mM of NaCl, 2.7 mM of KCl, 10 mM of Na_2_HPO_4_, 1.8 mM of KH_2_PO_4_ + 0.05% Tween-20, and *v/v* + 5% skimmed dry milk, *w*/*v*) for 1 h at room temperature. Then, the membrane was washed twice with 20 mL of PBST for 5 min under gentle agitation. After that, it was incubated with the anti-His(C-term)-HRP antibody (Invitrogen, Life Technologies) diluted 1:5000. For the Western blot immunoassay adapted from [21,22], a supplementary addition of convalescent sera from Tunisian patients infected with SARS-CoV-2, Wuhan strain, diluted 1:200 in PBS-T + 2.5% skimmed dry milk (*w*/*v*) overnight at 4 °C under gentle agitation, was also needed. Following this, three washing steps with PBS-T were carried out. For the detection of His-tagged S1RBD, a signal was detected directly using the Enhanced Chemiluminescence (ECL) kit (GE Healthcare). For the immunoblotting detection of anti-IgG SARS-CoV-2 antibodies, the membrane was incubated with the secondary peroxidase-conjugated anti-human IgG (HRP) (Invitrogen) (1:10,000) for 1 h at room temperature. The signal was then detected using the ECL kit.

The current work was carried out within national and international projects. The study protocol was approved by the national ethical committee at the Institut Pasteur of Tunis (references of ethical approval: 2021/02/I/LR16IPT and 2020/21/I/LR16IPT).

#### 2.2.5. In-House Indirect ELISA to Detect IgG Anti-S1RBD

Sera samples (Wuhan strain) were diluted at 1:200 or 1:400 in PBS-0.01% Tween 20 and incubated in 96-well plates (Nunc, MaxiSorp) coated with recombinant S1RBD protein (50, 100, or 200 ng per well). After incubation, the wells were washed, and the peroxidase-labeled anti-IgG antibody (1:8000) was added. After washing, the substrate solution (TMB, BD Biosciences, Franklin Lakes, NJ, USA) was added, and the plates were incubated for 10 min in the dark. After adding the stop solution (H_2_SO_4_ 2N, Sigma), the optical density (OD) values were measured at 450 nm and 630 nm.

## 3. Results and Discussion

### 3.1. Small-Scale Production of the His-Tagged S1RBD Spike Protein in Sf9 Cells

#### 3.1.1. Preliminary Assay

**Figure 1:**
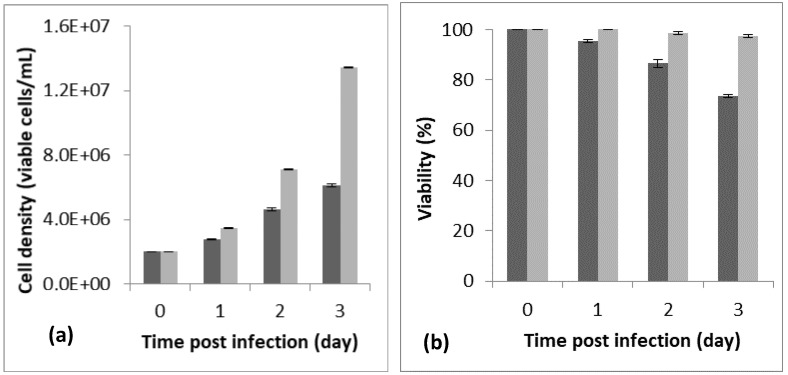
Monitoring of cell density (**a**) and viability (**b**) for 72 h post-infection (PI). The preliminary assay was carried out in a 125 mL baffled shake flask with a working volume of 20 mL, a cell density at infection of 2 × 10^6^ cells/mL, and an MOI of 5. The dark bar refers to the infected flask and the light bar to the blank (non-infected flask). The assay was repeated 3 times, and the bar errors indicate the mean standard deviation.

Cell density and cell viability, respectively, of 6.18 × 10^6^ cells/mL and 88% were reached 72 h post-infection, using a cell density at infection of 2 × 10^6^ cells/mL. The assay was carried out at an MOI of five, in a 250 mL baffled shake flask with a working volume of 20 mL (Figure 1). We noted that the best infection efficiency was obtained with cells in the active (better in the middle) exponential growth phase, which confirmed the literature. For example, Carinhas et al. explained that a new culture inoculated with cells from the middle exponential phase can achieve a slightly higher cell concentration and protein expression yields than one from the late exponential phase. That could be linked to the higher percentage of active cells (in phases S and G1) in the inoculum [23].

To further optimize the His-tagged S1RBD spike protein expression in the Sf9 cells, the effect of MOI, cell density at infection, and harvest time were studied.

#### 3.1.2. MOI Assay

**Figure 2:**
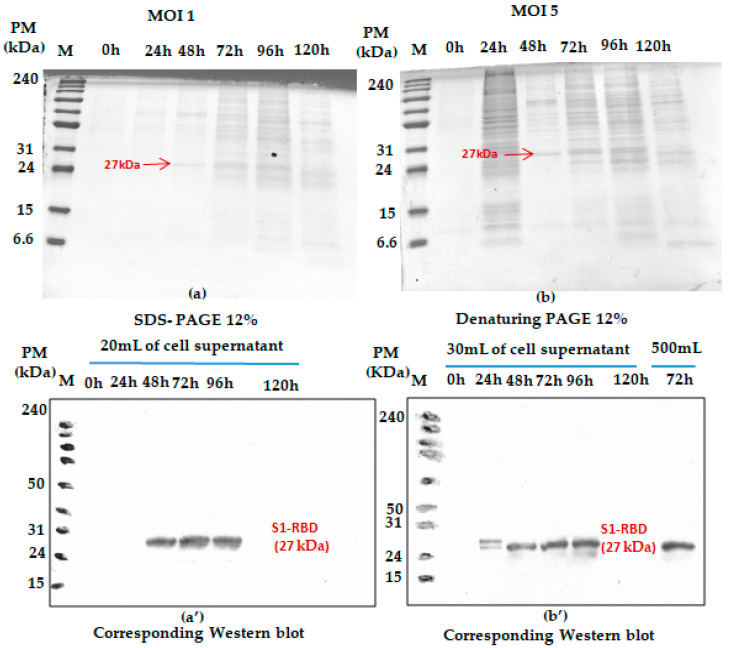
Monitoring S1RBD protein expression in Sf9 cells grown in Erlenmeyer flasks. The cells were infected with 2 × 10^6^ cells/mL at MOI 1 (**a**); corresponding Western blot (**a’**) and MOI 5 (**b**) corresponding Western blot (**b’**). Supernatants harvested at different times post-infection (tPI) were analyzed by Coomassie-stained 12% SDS-PAGE and Western blot. The working volume increase (from 20 to 500 mL) effect was also assessed in baffled shake flasks. Equal volumes were loaded onto the SDS-PAGE gel.

The effect of MOI on the recombinant His-tagged S1RBD expression (detection by Western blot analysis using anti-Histidine antibodies) was studied in 250 mL baffled shake flasks at different MOIs (we started with 1 and 5). As shown in Figure 2, S1RBD protein was detected in the culture supernatant only after 48 h PI and 24 h PI, respectively, at MOI 1 and MOI 5, to 96 h of post-infection, and no signal was detected at 120 h of post-infection (This was also confirmed in other assays such as Figure S4).

In addition, any significant difference (related to the S1RBD expression level at 72 h post-infection) was detected during the increase in working volume from 20 to 500 mL (Figure 2b’ only; this sample was only analyzed by Western blot). Such observation can be considered a good indicator for successful scaling up. Moreover, better protein expression was reached with an MOI of three. This refers to the band intensities of the corresponding Western blot results. A comparison was carried out with the protein (S1RBD) band intensity obtained at MOIs 1 and 5, and a fixed cell density at infection of 2 × 10^6^ cells/mL (Figure 2). As is known for the BEVS expression system, MOIs higher than unity should always be used to maximize protein expression yield [24,25].

It is of utmost importance here to mention that a low MOI, inferior to 1, is mainly used for virus amplification, as it allows a small subset of cells to be infected initially and to produce the budded virus. Thus, the infection of Sf9 cells at a low MOI is considered a key factor in successful virus amplification [26].

**Figure 3:**
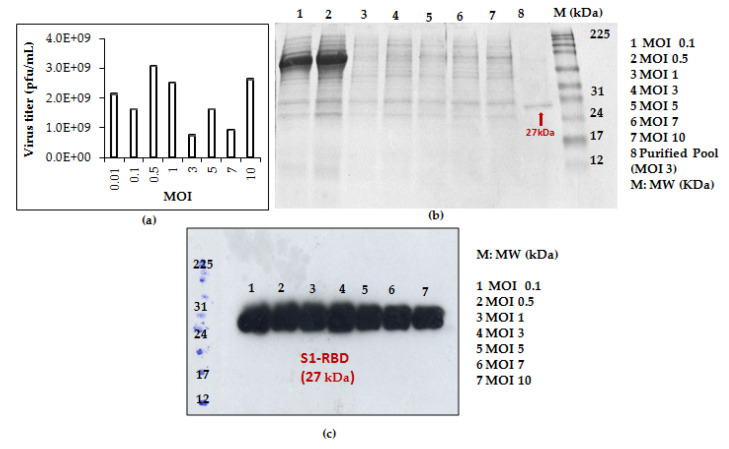
Recombinant baculovirus titer and S1RBD expression at different MOIs. The virus titer (**a**) as well as S1RBD expression profile 72 h post-infection using Coomassie-stained SDS-PAGE 12% (**b**) and Western blot (**c**).

This prompted us to analyze S1RBD expression at 72 h post-infection using additional MOI values: 0.01; 0.1; 0.5; 1; 3; 5; 7; 10. As presented in Figure 3a, a higher viral titer (3.08 × 10^9^ pfu/mL) was observed at low MOI values (0.01; 0.1; 0.5; 1). The experiments were carried out simultaneously in shake flasks at a fixed cell density at infection of 2 × 10^6^ cells/mL.

S1RBD was efficiently expressed (72 h PI) in the Sf9 cells at all the tested MOI levels (MOI 0.01 was only analyzed by virus titration), with slightly better expression at MOI 3 (Figure 3c). After purification, the protein yield of 4.3 mg and 1.4 mg S1RBD per liter of crude cell culture supernatant was achieved (Table S1 in supplementary data).

Some data analysis using Image J software, version 1.53t 24 August 2022. (see supplementary data Figure S5) confirmed the observations. Better band intensity levels were observed from 72 to 92 hPI.

In fact, optimal MOI depends on the cell line, the baculovirus, the medium, the mode of operation, and the physiological state of the cells. MOI affects protein productivity, the production of defective particles, and the time of exposure to proteases [15].

Based on protein yield after purification (line 8, Figure 3b and other optimization data), the MOI 3 was selected for the scale-up and further studies.

#### 3.1.3. Cell Density at Infection Assay

Cell density at infection is also an important parameter to consider while expressing proteins in the BEVS system.

**Figure 4:**
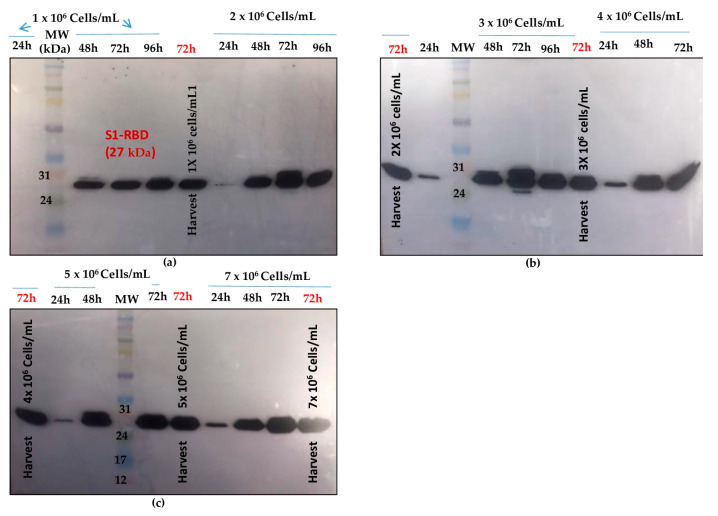
S1RBD expression profiles at different cell densities at infection (1 × 10^6^ and 2 × 10^6^ cells/mL (**a**); 3 × 10^6^ and 4 × 10^6^ cells/mL (**b**); 5 × 10^6^ and 7 × 10^6^ cells/mL (**c**), and a fixed MOI of 3 in shake flasks. Equal volumes were loaded onto the SDS-PAGE gel.

Shake flask experiments were carried out using different cell densities at infection. (1 × 10^6^, 2 × 10^6^, 3 × 10^6^, 4 × 10^6^, 5 × 10^6^, and 7 × 10^6^ cells/mL) at a fixed MOI of 3. As shown in Figure 4, better S1RBD expression levels were observed at cell densities of 2 × 10^6^ and 3 × 10^6^ cells/mL at 72 h post-infection.

Data analysis using Image J software (see supplementary data Figure S5) confirmed the observations.

Zhang et al. reported that MOI and cell density at infection (CDI) are correlated. They explained that higher productivity was obtained in cultures infected at higher CDI and MOI [27]. Indeed, studies that have addressed the combined effects of CDI and MOI on recombinant protein titers demonstrate that for each CDI, there is an optimum MOI for maximum protein production [28].

For scaling-up experiments (bioreactor cultures), we planned to use a minimum CDI of 3 × 10^6^ cells/mL and an MOI of three.

### 3.2. Scale-Up of the Optimized Process

Enhancement of SARS-CoV-2 spike RBD protein production has been extensively studied recently, e.g., in HEK293 cells, due to its high sensitivity in serological assays [5,27]. In the current work, we investigated the scalability of the process by producing S1RBD protein in larger working volumes of 500 mL and 5 L, respectively, in 2 L baffled shake flasks and a 7 L stirred bioreactor.

The shake flask cultures were infected at MOI 3 and a cell density at infection of 2 × 10^6^ cells/mL.

On the other hand, the bioreactor culture was initiated at a starting cell density of 0.5 × 10^6^ cells/mL. After 96 h (exponential cell growth phase), the cells reached 4 × 10^6^ cells/mL. They were infected at an MOI of 3. In the bioreactor experiments, cell density, cell viability, medium osmolarity, pH, and the S1RBD expression level were monitored up to 120 h post-infection.

**Figure 5:**
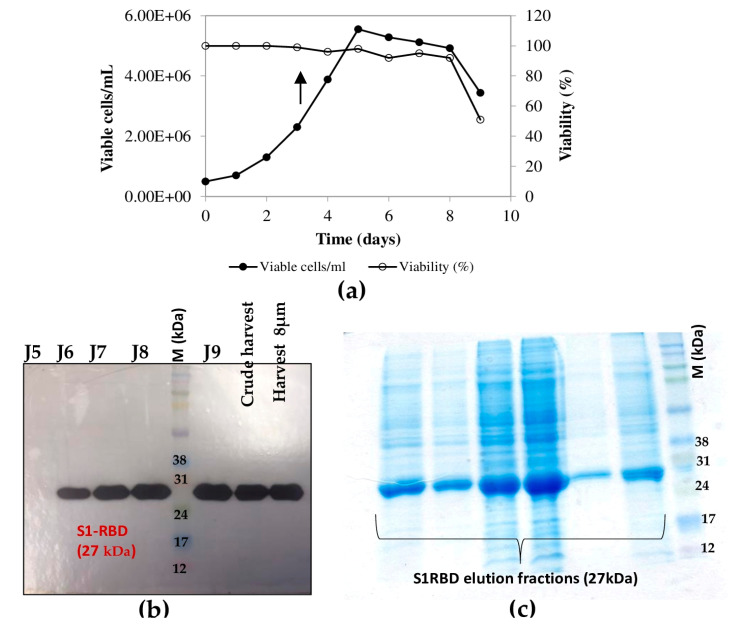
Growth curve of infected Sf9 cells and production of S1RBD in a 7 L bioreactor. Cell density and viability (**a**) (the arrow indicates the point of infection), and time course analysis of S1RBD expression in 7 L stirred bioreactor (**b**). One-step purification of S1RBD by affinity chromatography followed by Coomassie-stained SDS-PAGE 12% (**c**).

The highest cell density was 5.55 x 10^6^ cells/mL at 24 h post-infection (Figure 5a). The cell viability decreased to approximately 92-95% by 96 h post-infection and then dropped to 51% at 120 h post-infection (Figure 5b). Interestingly, increased expression of S1RBD was observed in the bioreactor at 96 h and 120 h post-infection, compared to the shake flask experiments where respectively low- and no-expression levels were detected at 96 h and 120 h PI (Figure 5c). 

As described by Thompson et al. [29], the time of harvest may depend on the MOI.

### 3.3. Downstream Process

**Figure 6:**
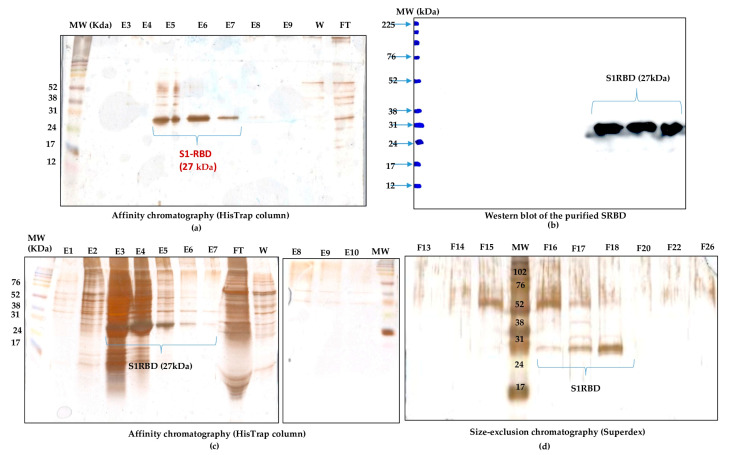
Purification of S1RBD and its detection by Western blot. (**a**) One-step purification of S1RBD by affinity column followed by silver-stained SDS-PAGE 12%: E5, E6, and E7 correspond to the eluted S1RBD fractions with the expected apparent molecular weight of 27 kDa. (**b**) Western blot analysis of the purified fractions. Two-step purification of S1RBD by affinity column (**c**) followed by size exclusion chromatography (**d**). Analysis was performed using silver-stained SDS-PAGE 12%. MW indicates the molecular weight of the protein marker.

The purification of the S1RBD started with the cell harvest. The crude culture supernatant was then clarified by centrifugation (for low volumes) and 8µm or depth filters (for large volumes). Then, TFF was performed (buffer exchange and concentration, as described in the Materials and Methods section). The culture supernatants were then concentrated at least tenfold to reach final volumes of 150 mL and 30 mL, corresponding to total protein concentrations of 0.32 mg/mL and 1.11 mg/mL, respectively (Table S1). Afterward, the S1RBD protein, tagged at its C-terminus with hexa-histidine, was purified in a two-step purification using a His-Trap FF column with a high purity of more than 95%, estimated on silver-stained SDS-PAGE 12%, to allow more sensitive detection of impurities. Finally, polishing and imidazole salt elimination were performed with Superdex Peptide or PD10 columns for the small volumes (Figure 6a, line E6). One-step purification followed by Vivaspin buffer exchange was also successful.

The eluted fractions were then pooled, and the protein levels were quantified using a colorimetric assay (Bradford). The one-step purification protocol results in an average yield of 4.45 ± 0.15 mg per liter of crude cell supernatant (Table S1), which is at least twofold higher than the yield obtained in HEK293SF cells using non-viral and viral production approaches [5]. The purified S1RBD was analyzed by Western blot (Figure 6b). A lower purification yield of S1RBD protein was also obtained using the one-step HisTrap FF column purification (Figure 6c, columns E3, E4, and E5). Therefore, the elution fractions from E3 to E10 were pooled, concentrated (MWCO 10 kDa) using Vivaspin centrifugal concentrators, and quantified. A volume of 0.5 mL, with a protein concentration of 1.55 mg/mL, was injected into the AKTA protein purification system for a second purification step by size-exclusion chromatography. Thus, a higher purification efficiency was observed after analysis of silver-stained SDS-PAGE 12% (Figure 6d, lane F18). The two-step purification protocol leads to an average yield of 1.4 mg per liter of cell culture (Table S1), which is approximately 1.5 times higher than the yield obtained in the HEK293SF cells [5]. The lower yield obtained with the two-step purification protocol could be explained by the protein loss during the procedure.

Interestingly, for clarified (8µm membrane) 7L culture harvests, the single-step purification method results in a yield of at least 70 mg S1RBD per liter of crude supernatant. The corresponding band (27 kDa) was detected by SDS-PAGE 12% and confirmed by Western blot (Table S1 and Figure 4d). The obtained production yield was about 50 times higher than the yield obtained using the 2 L shake flasks. This proves the effectiveness of the upstream and downstream processes.

Using different cell expression hosts, Expi293F cell line, higher yields were obtained for RBD (approx. 90 mg/L) 3 days post-transfection. Such yield was only achieved by HEK293-E6 cells when the culture was extended two extra days (harvesting on Day 5 post-transfection) [30].

**Figure 7:**
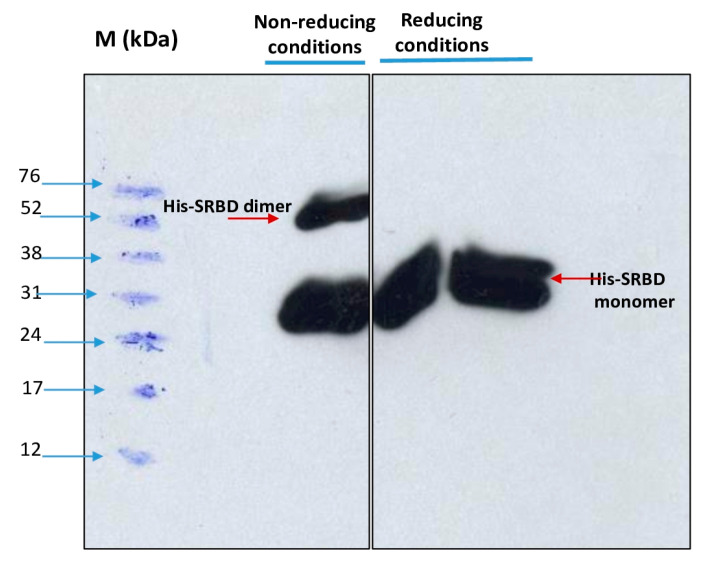
Dimerization analysis of purified S1RBD on SDS-PAGE 12% under non-reduced (**left**) and reduced (**right**) conditions.

Dimerization of the purified S1RBD was verified by non-reducing SDS-PAGE analysis, and as expected, two bands appeared in the line in which the protein sample was not reduced with 2-mercaptoethanol: a dominant band with a molecular weight of 27 kDa and a less intense band with a Mw of about 54 kDa (Figure 7).

In the control lines where the samples were reduced in the presence of 2-mercaptoethanol, a distinct single band was observed. These results corroborate those reported by Tee et al. [31], who showed two bands of the same molecular weight for the unreduced RBD protein produced in HEK cells. The S1RBD sequence (residues 319–541) has four disulfide bonds (C336-C361, C379-C432, C391-C525, and C480-C488) and a free C538 that may be involved in intermolecular dimerization [9].

### 3.4. Immunoassays of the His-Tagged S1RBD Spike Protein

**Figure 8:**
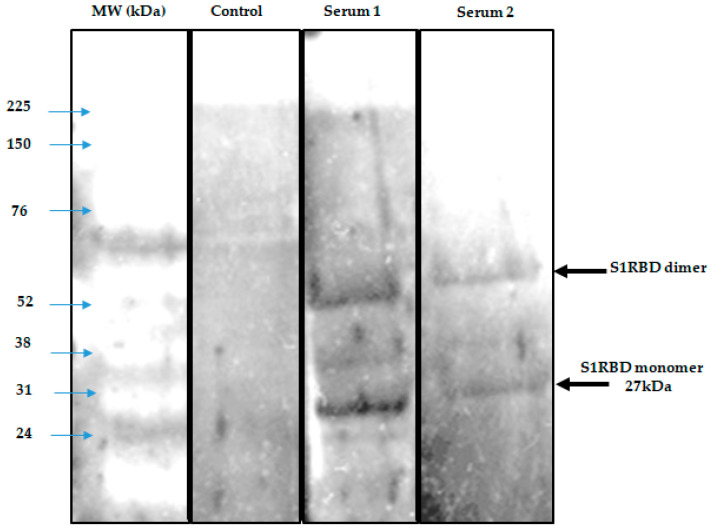
Development of a Western blot assay to detect seropositivity for SARS-CoV-2 infection. A negative control and 3 sera were used in duplicate.

To demonstrate the antigenicity of S1RBD against anti-IgG-SARS-CoV-2 antibodies, a Western blot was developed using Tunisian convalescent COVID-19 patient sera. Indeed, the anti-IgG antibodies recognized the purified S1RBD protein, and Western blot analysis clearly showed a band with a molecular weight of 30 kDa corresponding to S1RBD in both sera 5 and 6 of the convalescent COVID-19 patients, although the band was more pronounced in patient 5, which could be explained by a higher IgG anti-SARS-CoV-2 antibody titer (Figure 8).

Indeed, it has been reported that the recombinant SRBD protein produced in Sf9 insect cells has an excellent antigenicity with convalescent COVID-19 patient sera [13,16]. We also observed the presence of a second band with a higher molecular weight of about 52 KDa, which could correspond to the dimer of S1RBD, despite the reduced conditions used and the slight difference in molecular weight compared to the S1RBD dimer (~60 kDa) tested under the non-reduced conditions (Figure 7) [32]. BEVS expression vector system-expressed S1RBD produced in SF9 cells has excellent antigenic potential, as reported by Li et al. [14].

It has also been shown that RBD dimerization induced high levels of RBD-binding and SARS-CoV-2-neutralizing antibodies in both mice and non-human primates [33].

### 3.5. Development of Indirect ELISA for Anti-S1RBD IgG

**Figure 9:**
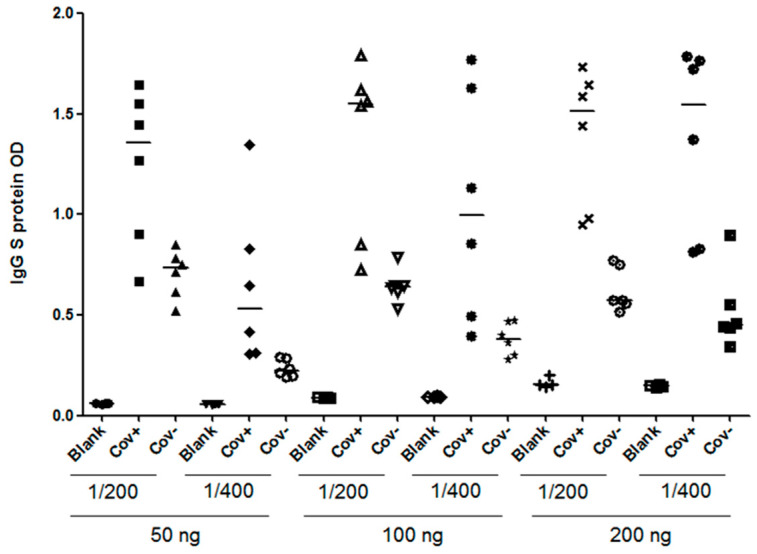
Optimization of an indirect ELISA utilizing S1RBD antigen of SARS-CoV-2. COVID-19 patients’, confirmed using RT-PCR (*n* = 6), and pre-pandemic healthy controls’ (*n* = 6) sera were tested for anti-S1RBD IgG antibodies by varying several parameters. Plates were coated with different concentrations of S1RBD recombinant protein (50 ng, 100 ng, or 200 ng) and sample dilutions (1/200 or 1/400); Cov+; COVID-19 patient, Cov-; Pre-pandemic healthy control and blank: PBS.

In order to achieve the most effective assay conditions, it was crucial to establish an in-house protocol that provided an adequate amount of antigen to capture antibodies while minimizing non-specific background signals. Therefore, we initially coated plates with the recombinant protein S1RBD across a concentration range (from 50 to 200 ng per well). Subsequently, the serum samples were added at various dilution ratios (1:200 or 1:400) (Figure 9). Our optimization process was guided by two primary criteria: achieving the highest OD ratio between the positive and negative samples, and attaining the lowest OD ratio between the negative samples and the blank control.

In conclusion, the optimized working conditions were as follows: 100 ng per well for coating and a sample dilution of 1:400. The optimized assay was validated and cross-validated as described by Benabdessalem et al. [34], and the performance of the anti-S1RBD ELISA was higher, with a sensitivity of 95% and a specificity of 93%.

The purified S-RBD protein was also used to perform national and African seroprevalence studies [34,35].

While this coronavirus outbreak continues to spread, many studies have focused on conserved epitopes that may allow structure-based design, not only of a SARS-CoV-2 vaccine but also of cross-protective antibody responses against future coronavirus epidemics and pandemics [36].

## 4. Conclusions

In this study, the entire upstream and downstream process for the production of the RBD spike S1 protein of SARS-CoV-2 was characterized using Sf9 insect cells using the baculovirus vector expression system (BEVS). A purification yield higher than 4 and 70 mg S1RBD per liter of crude culture supernatant was achieved, respectively, in shake flasks and a stirred bioreactor. We demonstrated that this expression system was efficient for the overproduction of immunologically active S1RBD towards the IgG SARS-CoV-2 antibody, with a high potential for use in serological assays such as ELISA. These optimized production processes, using the baculovirus–insect cell system, will be of interest in other studies aimed at the overproduction of recombinant proteins for therapeutic or diagnostic purposes.

The current results of protein production using insect cells–BEVS were obtained in a bioreactor operated in batch mode. The MOI and CDI were selected after small-scale testing in shake flasks. The BEVS is widely used for the manufacture of biologics, including vaccines for humans and animals, therapeutics, gene therapy vectors, and bio-pesticides.

This work was directed by national priorities during the COVID-19 outbreak. The choice of the BEVS expression system was based on the availability of know-how and cost-effectiveness.

## Data Availability

Not applicable.

## Supplementary Materials

The following supporting information can be downloaded at: https://www.mdpi.com/article/10.3390/tropicalmed8110501/s1, Figure S1: Map of the recombinant pFASTBac-S1RBD vector.; Figure S2: Schematic representation of the preparation of the recombinant bacmid (a) and transfection of Sf9 cells (b); Figure S3: Schematic representation of the amplification process of recombinant baculovirus (a) and S1RBD protein (b); Figure S4: S1RBD expression in Sf9 cells. Total proteins from the culture supernatant were analyzed at different time points post-infection (tPI) with MOI 1 (a) and MOI 5 (b). The assays were analyzed by Coomassie-stained SDS-PAGE 12% and Western blot. The cell density at infection was 2 × 10^6^ cells/mL in a baffled shake flask. Equal amounts of total proteins (10 μg) were loaded onto the SDS-PAGE gel, Figure S5: band density evaluation using the Image J software, and Table S1: Supplementary data related to the downstream process.
