# Supplementary material for: Development of an Optimized Process for Functional Recombinant SARS-CoV-2 Spike S1 Receptor-Binding Domain Protein Produced in the Baculovirus Expression Vector System
Source: Trop Med Infect Dis. 2023 Nov 16;8(11):501. doi: 10.3390/tropicalmed8110501 (PMC10674791; doi:10.3390/tropicalmed8110501)
Supplement: Supplementary file 1 [file tropicalmed-08-00501-s001.zip › tropicalmed-2596256-supplementary.pdf]

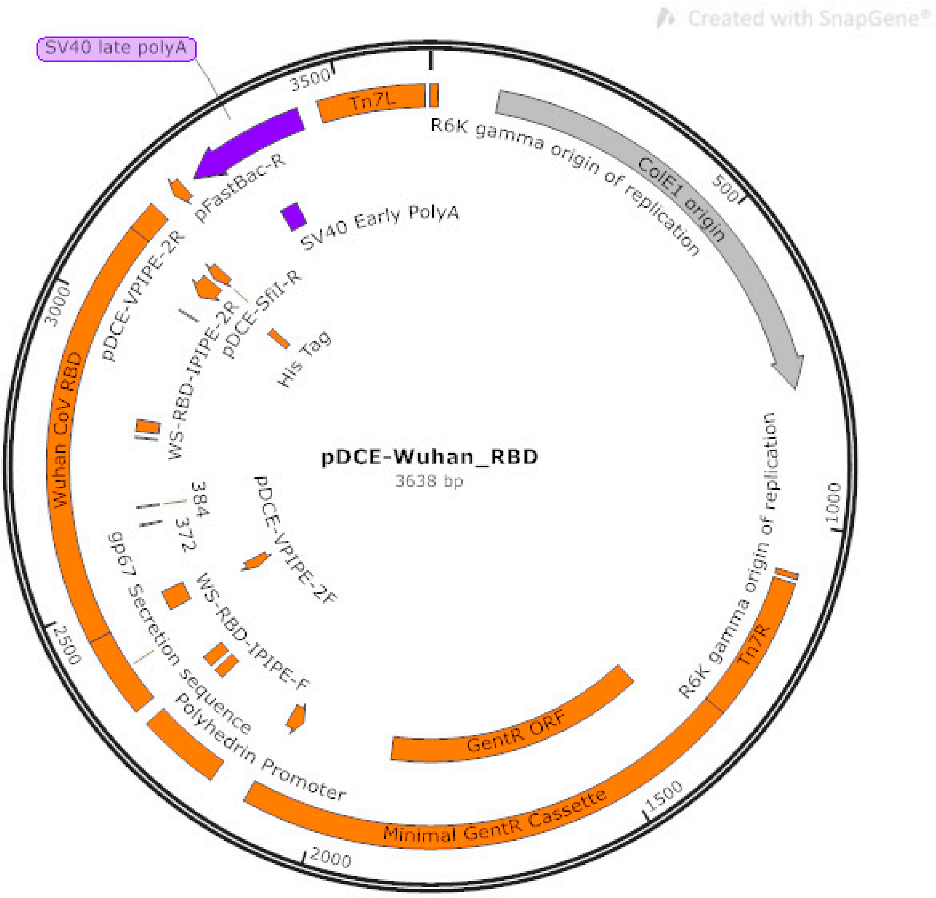

Figure S1. Map of the recombinant pFASTBac-S1RBD vector.

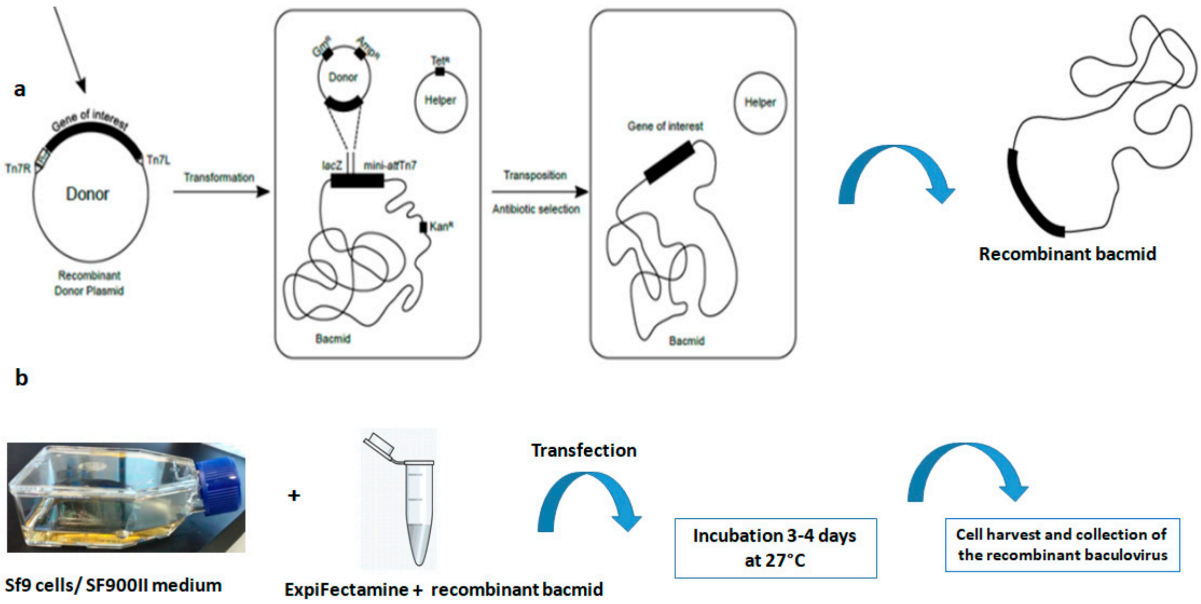

Figure S2. Schematic representation of the preparation of the recombinant bacmid (a) and transfection of Sf9 cells (b).

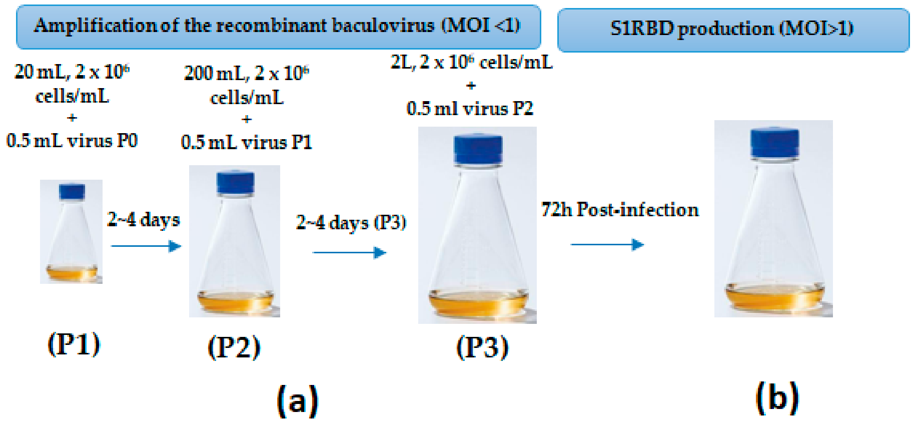

Figure S3. Schematic representation of the amplification process of recombianant baculovirus (a) and S1RBD protein (b).

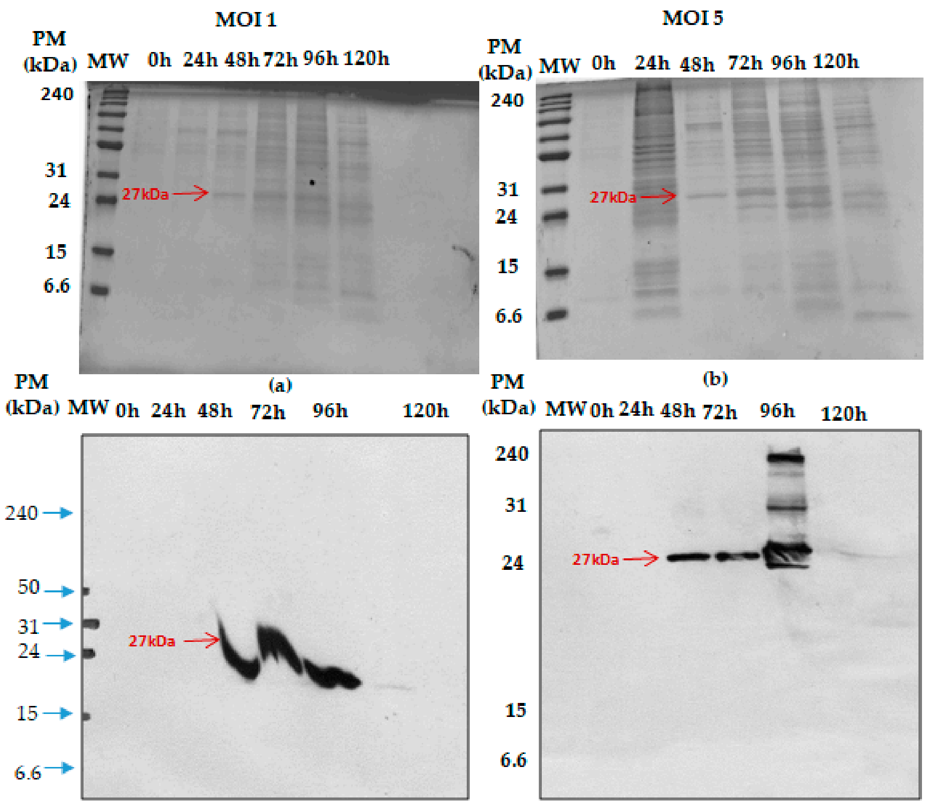

Figure S4. S1RBD expression in Sf9 cells. Total proteins from the culture supernatant were analysed at different time points post infection (tpi) with MOI 1 (a) and MOI 5 (b). The assays were analysed by Coomassie-stained SDS-PAGE 12% and Western blot. The cell density at infecton was 2 x 10^6 cells/mL in buffled shake-flask. Equal amounts of total proteins (10 μg) were loaded onto the SDS-PAGE gel.

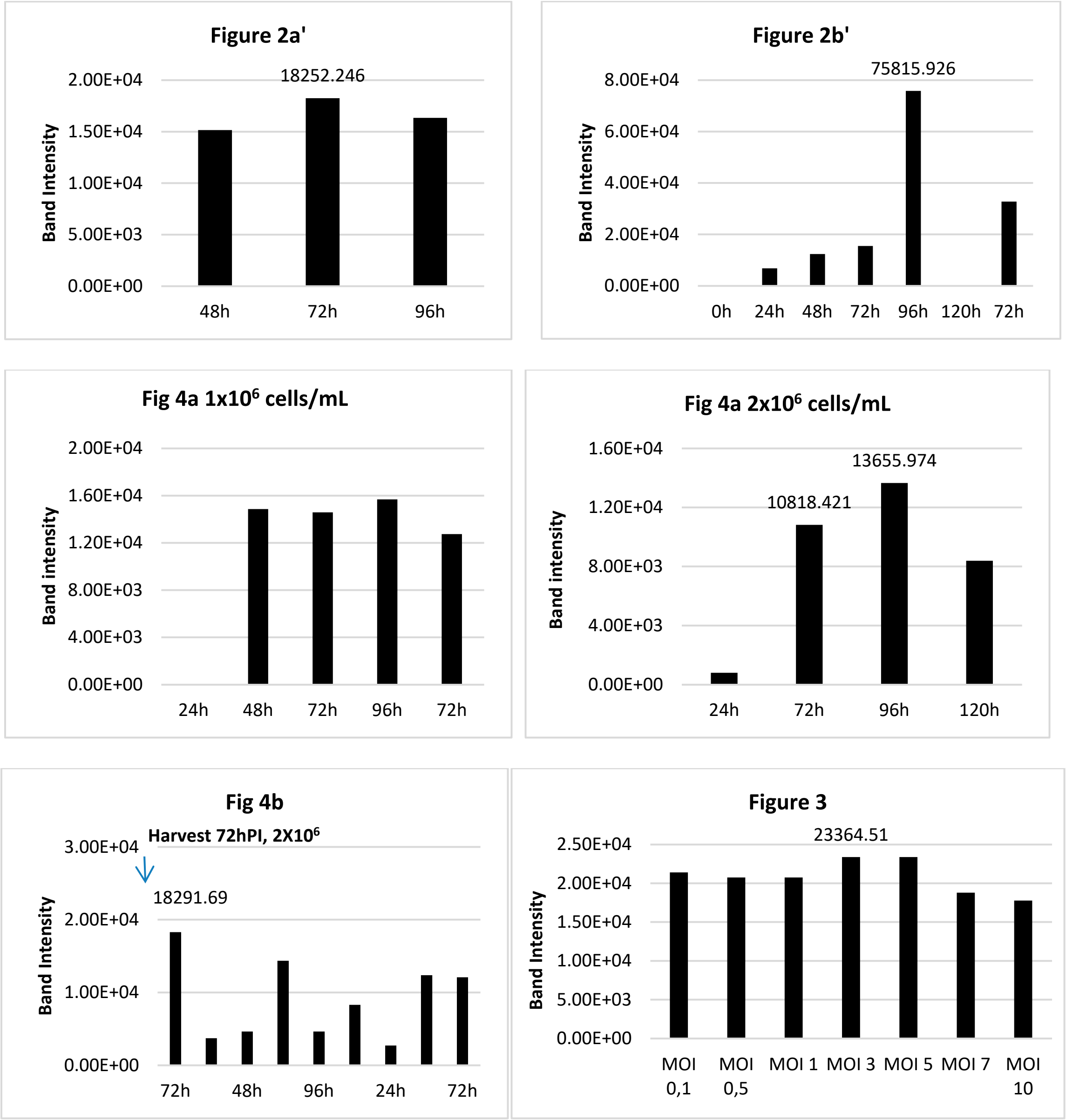

Figure S5. Examples of band density evaluation using the software "image J"

Table S1. Supplementary data related to downstream process

Culture Conditions	Purification method	Volume[a] (mL)	Total quantity of S1RBD (mg/L of crude supernatant)[b]
**Shake-flasks**	Ni-NTA affinity column Followed by gel filtration	150	4.45±0.15
	Ni-NTA column + Superdex increase column	30	1.69±0.4
**7L-bioreactor**	NTA column Followed by gel filtration	400	70±8

[a]Volume after buffer exchange and concentration (after TFF)
[b]The results are expressed as mean ± standard deviation (n = 3)
